# Decorated graphene oxide with gold nanoparticles as a sensitive modified carbon paste electrode for simultaneous determination of tyrosine and uric acid

**DOI:** 10.1038/s41598-023-44540-6

**Published:** 2023-10-15

**Authors:** Elahe Garazhian, Majid Kalate Bojdi, Mohammad Behbahani

**Affiliations:** 1 Department of Chemistry, Faculty of Science, University of Birjand, Birjand, South Khorasan Iran; 2 Department of Chemistry, Faculty of Science, Shahid Chamran University of Ahvaz, Ahvaz, Iran

**Keywords:** Medical research, Chemistry, Materials science

## Abstract

It is presented here as a simple, selective, rapid, low-cost, with a wide linear range method to simultaneously determine tyrosine and uric acid using a modified carbon paste electrode decorated with graphene oxide and gold nanoparticles (GO/AuNPs/MCPE). In order to characterize and evaluate the morphology and constituents of the nanostructures, X-ray diffraction spectroscopy, Transmission electron microscopes, Dynamic light scattering, Zeta potential, electrochemical impedance spectroscopy, and Voltammetry were employed. The current response on the surface of the modified electrode had a dynamic linear range relationship in the concentrations of 0.14–340.00 µmol L^−1^ and 0.06–141.00 µmol L^−1^ for tyrosine and uric acid, respectively, and the method detection limit (MDL) was 0.0060 µmol L^−1^ and 0.0037 µmol L^−1^, respectively. This modified electrode provided high stability, sensitivity, and acceptable reproducibility for voltammetric measurements of tyrosine and uric acid simultaneously in a biological matrix.

## Introduction

Uric acid's chemical formula is C_5_H_4_N_4_O_3_, a heterocyclic compound composed of carbon, nitrogen, oxygen, and hydrogen. Uric acid is a raw waste material produced as a by-product of purine decomposition. An individual with a healthy kidney system excretes uric acid from the blood into the urine. Due to the fact that some kidney diseases are diagnosed based on the level of uric acid in the blood, the determination of uric acid is crucial for the diagnosis of kidney diseases. A high uric acid level in the blood can cause gout, a painful disease caused by the crystallization of the acid. A high uric acid level in the body can also lead to uremia, leukemia, and pneumonia^1^. Uric acid is an effective reducing agent and a robust antioxidant that makes up approximately half of the antioxidant capacity of human blood plasma. A majority of the uric acid produced by the body is excreted in the urine by the kidneys every day. Increased serum uric acid levels or hyperuricemia are caused by kidney function declining by 5–25%^2,3^.

Tyrosine is an amino acid with the chemical formula C_9_H_11_NO_3_, referred to as 2-amino acid 3-(hydroxyphenyl) propanoic acid. Tyrosine is an essential amino acid used by the body to synthesize proteins and is one of the 20 amino acids found in the human body. It is a water-soluble amino acid with low solubility. According to Wikipedia, tyrosine takes its name from the Greek word (Tyri), which means cheese, as a German chemist named Liebing was the first to isolate tyrosine from casein protein in cheese in 1846^4^. Dopamine, epinephrine, and norepinephrine are neurotransmitters produced as a result of protein synthesis in the human body. Tyrosine is also called para-hydroxyphenylalanine because it is broken and converted into tyrosine by the oxidation of phenylalanine after entering the body by an enzyme called phenylalanine hydroxylase^5^. It is common to find tyrosine in natural foods; however, its low levels are associated with low blood pressure, low body temperature, albinism, phenylketonuria, alkaptonuria, and hypothyroidism. A person who cannot meet their body's need for this substance through natural food sources may be at risk of its dangerous effects^6^. Due to the accumulation of phenylalanine in different tissues, phenylketonuria disease, which is a disorder of phenylalanine no acid metabolism, causes brain damage and mental retardation. A sample of a tyrosine supplement is being produced and is available for you to try. Typically, a normal individual requires 5–7 g of this amino acid per day. Despite the fact that this substance is not harmful to the body, its excessive consumption poses a threat to health, and an overdose of this amino acid can lead to an increase in blood pressure and skin problems. Natural sources of this amino acid include meat, dairy products, eggs, carrots, and bananas^7^. A biological fluid such as serum or urine generally contains tyrosine and uric acid. The biological environments of blood serum and urine are very complex and include a variety of compounds. The determination and control of these compounds are crucial and necessary in the diagnosis and treatment of many diseases. This is because changes outside their natural range can disrupt the stability of the internal compounds of the body, causing disorders in human health. There is an important need to establish methods that make it possible to measure these essential compounds simultaneously, like tyrosine and uric acid, since their changes are highly dependent on each other^8^.

The modified electrode showed acceptable repeatability and reproducibility parameters in the studies conducted. Electrode specificity was significantly increased, and electron transfer kinetics were improved on the electrode surface modified during the electrochemical oxidation of tyrosine and uric acid, which resulted in a significant increase in the voltammetric response compared to the bare carbon paste electrode. High-performance chromatography^9,10^ and spectrometry^11,12^ Even though this method operates properly, it has disadvantages such as a high price, a long analysis time, and a requirement to prepare the difficult and complex sample construction, making it not suitable for daily measurements. Despite this, the methods for measuring uric acid and tyrosine still have disadvantages. Due to their high sensitivity, simplicity, speed, affordability, and lack of need for sample preparation, electrochemical techniques have been highly regarded today^13–15^. They have been reported to determine tyrosine and uric acid^16–18^. The yellow color of gold is due to the reflection of blue light at the end of the spectrum, but the size of the particles becomes smaller than the wavelength of the reflection as the size of the particles decreases. In this situation, the interaction between gold and light will cause electronic oscillations followed by surface plasmon resonance^19^. The tendency and attachment of sulfur-containing groups to the surface of gold particles have attracted many researchers’ attention to using gold nanoparticles in many types of research^20^. Gold nanoparticles have received considerable attention in recent years. In order to analyze biological materials, nanoparticles as modifiers have attracted the attention of many researchers due to their high surface area, good biocompatibility, relatively good conductivity, and physical and chemical properties^21,22^.

## Experimental

### Materials and instruments

All electrochemical measurements were performed using a potentiostat/galvanostat (Vertex, Ivium, Eindhoven, Netherlands) connected to a personal computer and controlled by IviumSoft 2.5 software. A three-electrode setup was used for performing experiments in a 50 mL glass cell. A modified carbon paste electrode decorated with graphene oxide and gold nanoparticles (GO/AuNPs/MCPE) as a working electrode, a platinum electrode as an auxiliary electrode, and a saturated calomel electrode (SCE) as a reference electrode (manufactured by Azar Electrode Company) were used for voltammetric measurements. In this research, the working electrode was made separately. All the potentials are reported relative to the reference electrode. Also, a pH/mV meter was used to measure pH.

Tyrosine, uric acid, sodium hydroxide, phosphoric acid, graphite powder, ecosan, dipotassium hydrogen phosphate, and potassium dihydrogen phosphate were supplied from Merck, Germany.

### Preparation of the modified electrode

The electrode was prepared by mixing gold nanoparticles-modified graphene oxide (8% weight percent), graphite powder, and ecosan (62:30% weight percent) for 20 min at 40 °C. As a next step, it was transferred to a Teflon tube with an inner diameter of 5 mm and an outer diameter of 2 mm, and its connection was established via a copper wire located in the middle of the tube.

### Synthesis of nanoparticles

We obtained GO and Au NPs from local suppliers in Iran (Nanosany Co. and Iranian Nanomaterials Co.). It is essential for the preparation of Nanofluids that carbon materials are dispersed in a base fluid. Because of the van der Waals interaction between carbon sheets and the large aspect ratio, dispersing carbon materials in aqueous solutions is challenging. Under normal conditions, graphene Nanosheets cannot be dispersed in water due to their hydrophobic nature^23,24^. In order to prepare hydrophilic graphene Nanosheets (graphene oxide), COOH functional groups are used to functionalize graphene Nanosheets, and this results in the enhancement of the stability of the Nanofluids prepared^25^. Graphene oxide gold nanoparticles/water hybrid Nanofluid (GO/Au NPs/water) were prepared using a two-step procedure in our study^26–29^. By using suitable dispersion approaches, the NPs that were separately generated were dispersed in water. The nanomaterials (GO and Au NPs) were mixed with distilled water (DW). A stable homogeneous hybrid Nano fluid was prepared by ultrasonic homogenizer (UP400S, Hielscher GmbH; 400 Watts and 24 kHz, 5 min) with specific concentrations according to response surface methodology. It was prepared by preparing 1000 ml of gold nanoparticle solution with a concentration of 100.0 ppm (100 mg gold nanoparticle in 1000 ml water) and then dilution of the first solution (100 ppm) to 100 ml with distilled water to prepare Au NPs with a concentration of 50.5 ppm. A concentration of 1.0 ppm of Au NPs was then prepared by diluting 1 ml of the first solution (100 ppm) with 100 ml of distilled water.

### Characterization of the synthesized substrate

Material's properties depend on the properties of its constituents, NPs, and the base fluid, due to its content and nature. Therefore, it is important to have a thorough understanding of the properties of NPs. In ambient conditions, an X-ray powder diffractometer with a Cu Ka radiation source at 1.54 angstrom was used to study the crystal structure of NPs. In the low angle range of 1°–80°, the intensity of diffracted X-rays from the GO nanoparticles is plotted as a function of angle. These results provide information regarding the characteristics of the X-ray wave and the periodic ordering of the sample crystals. Based on the XRD pattern, there is only one peak with a constant intensity at an angle 2Ɵ = 10.46^30^.

**Figure 1:**
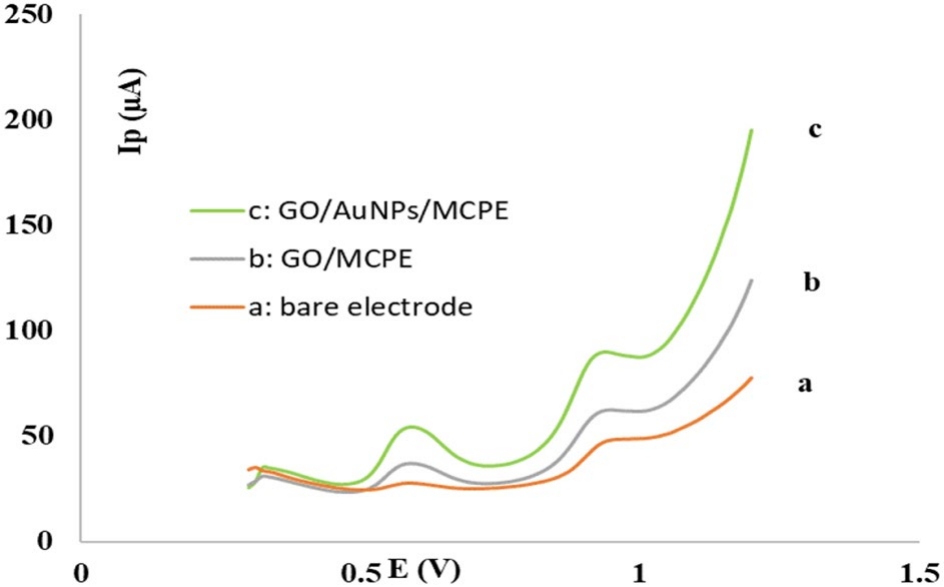
Electrochemical voltammogram of SWV technique of bare (**a**) GO/MCPE (**b**), and GO/AuNPs/MCPE (**c**) electrode in the presence of uric acid solution (with concentration 19.05 µmol L^−1^) and tyrosine (with concentration 23.2 µmol L^−1^) in 0.2 mol L^−1^ phosphate buffer solution and 0.1 mol L^−1^ potassium chloride at pH 2.0. Electrochemical condition: pre-potential of 0.42 mv applied for 10 s, used electrodes (working electrode: modified carbon paste electrode, reference electrode: SCE, auxiliary electrode: platinum wire), temperature: 25 °C.

**Figure 2:**
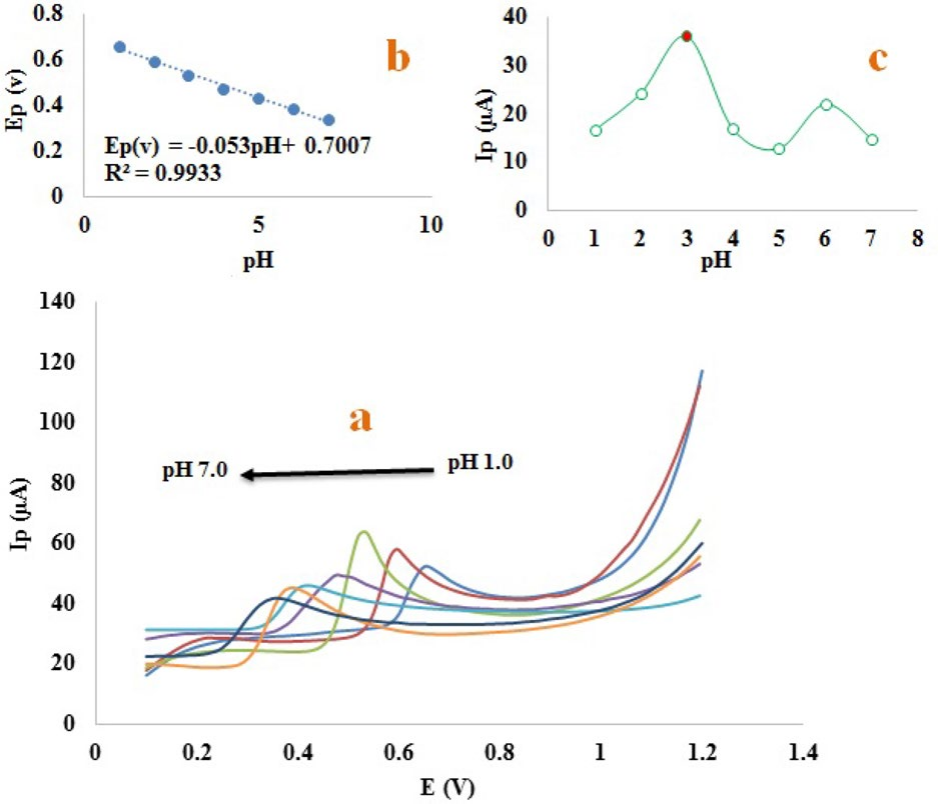
LSV voltammogram of uric acid at concentration of 14.15 µmol L^−1^ at different pHs, ranging from 1.0 to 7.0 at the surface of the modified electrode (**a**), E_p_ versus pH (**b**), Ip versus pH (**c**) in 0.1 mol L − ^−1^ buffer phosphate solution with a scan rate of 100 mv/s, used electrodes (working electrode: modified carbon paste electrode, reference electrode: SCE, auxiliary electrode: platinum wire), temperature: 25 °C.

Each peak's angle is determined by its distance from the platelet and its intensity by the order in which the atoms are arranged on the platelet. Based on the results, the developed sample meets all specifications, as only a single peak is observed with a specific intensity. The Transmission electron microscope (TEM) results (Fig. 1S) for the gold nanoparticles show very similar sizes, with an average diameter below 15 nm (the figure is not shown). In the use of Nanofluids, the primary parameter is the assessment of the dispersion of the NPs in the water. Zeta potential is the index of the surface charge of NPs that affects this factor. A significant value should be present for Nanofluids (stable colloids) due to the strong electrostatic repulsion that occurs between the NPs. In a Nanofluid with small stability, NPs would have weak repelling forces, and aggregation would occur with a low zeta potential index as a result of collisions between NPs. There is a general consensus that Nanofluids with zeta potentials above 30 mV are stable nanofluids, and nanofluids with zeta potentials below 20 mV are unstable nanofluids. The mean zeta potential of the developed hybrid Nano fluid was 52.6 mV Fig. 2S-a,b. Additionally, gold nanoparticles are observed as dark dots with an average diameter of 15 nm on a light-shaded substrate equivalent to planar graphene oxide sheets. Due to the sheet-like structure of graphene-based nanocomposites, low-intensity peaks in different-size regions have been commonly observed in previous research investigating graphene-based nanocomposites^31–34^.

## Results and discussion

To study the electrochemical activity of GO/AuNPs/MCPE compared to the unmodified electrode, square wave voltammetric (SWV) responses in the presence of uric acid and tyrosine solution with concentrations of 19.05 µmol L^−1^ and 23.2 µmol L^−1^ in 0.2 mol L^−1^ phosphate buffer solution with pH 2.0 and prepotential of 0.42 mv was investigated (Fig. 1).

The voltammetric response of the unmodified electrode is shown in Fig. 1a, which shows how the electrode reacts to the solution containing analytes. It is noted that the electrode's response to analytes is relatively low, and a peak of low current is observed, which indicates that it is not efficient. The voltammogram in Fig. 1b,c illustrates the response of the GO/MCPE and GO/AuNPs/CPE, indicating that the peak current of the modified electrode with GO/AuNPs is increased compared to the voltammogram in Fig. 1a,b. An investigation of the effects of different factors on the simultaneous determination of tyrosine and uric acid was conducted in order to optimize and obtain the best possible response of the electrode.

### The effect of pH

**Figure 3:**
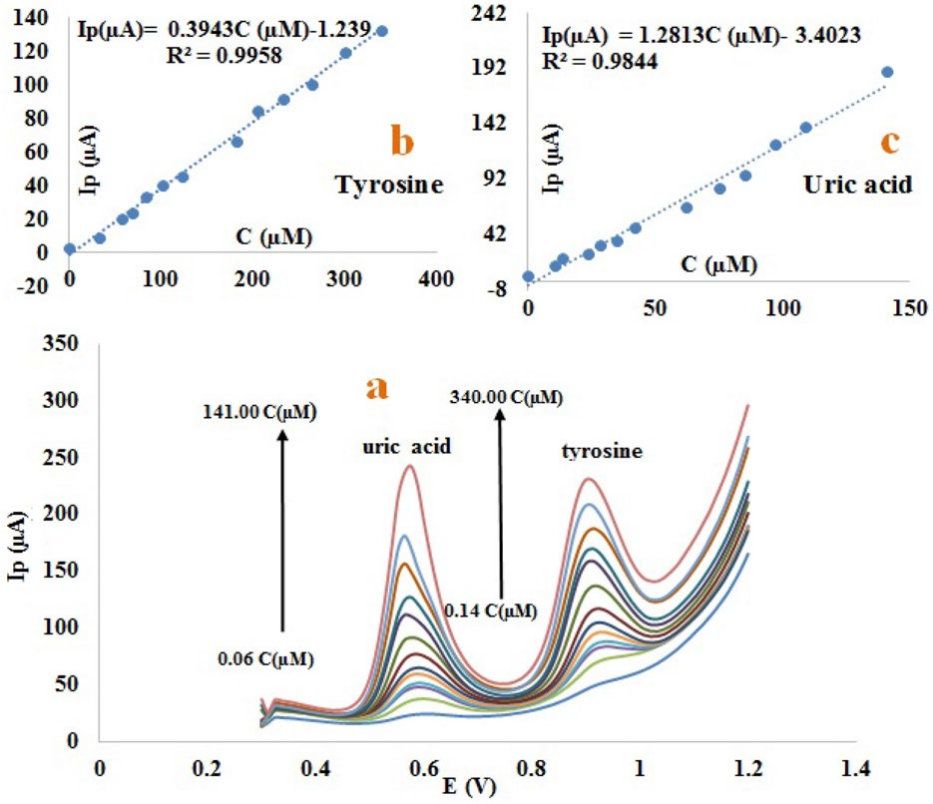
SWV of different concentrations of uric acid(0.06, 10.49, 13.7, 23.4, 28.5, 34..6, 42.1, 62.1, 75.4, 85.5, 96.9, 109.0, 141.0 µM) and tyrosine(0.14, 33.0, 56.7, 68.9, 83.7, 101.0, 123.0, 182.0, 206.0, 233.0, 265.0, 300.0, 340.0 µM) on the surface of the modified electrode (**a**), the linear calibration curve of peak current versus different concentrations of tyrosine solution (**b**), the linear calibration curve of peak current versus different concentrations of uric solution Acid (**c**), in 0.2 mol L^−1^ phosphate buffer solution and 0.1 mol L^−1^ potassium chloride, pH 2.0. Electrochemical condition: pre-potential of 0.42 mv applied for 10 s, used electrodes (working electrode: modified carbon paste electrode, reference electrode: SCE, auxiliary electrode: platinum wire), temperature: 25 °C.

The pH of the environment is one of the most important factors in electrochemical investigations and can have a significant impact on electrochemically determining tyrosine and uric acid oxidation currents. In this analysis, the effect of pH on peak potential is analyzed to determine the protons versus electrons ratio. Therefore, the effect of pH on uric acid and tyrosine oxidative behavior was evaluated separately by linear sweep voltammetry (LSV) technique in 0.1 mol L^−1^ phosphate solution for the modified electrode at concentrations of 14.15 µmol L^−1^ and 48.70 µmol L^−1^ in the pH range of 1.0–7.0. Figure 2 and Fig. 3S illustrate the results.

The pH affects the current and peak potential of uric acid, as shown in Fig. 2b,c. Equation (1) indicates a negative shift in anodic peak potential with an increase in pH, which indicates deprotonation during oxidation is facilitated at higher pH, as indicated by the slope of − 0.053 V. According to the slope value, the same number of protons and electrons participate in the tyrosine oxidation reaction, which is consistent with previous studies^35,36^.

1 $$ {\text{Epa}}\left( {\text{V}} \right) = - 0.0{53}\;{\text{pH}} + 0.{7}00{7}\quad \left( {{\text{R}}^{{2}} = 0.{9933}} \right) $$

As can be seen from Fig. 3S-b,c tyrosine's response current and peak potentials are both affected by pH. As pH rises in the buffer solution, the anodic peak potential values decrease, indicating the participation of protons in the tyrosine oxidation process. Equation (2) shows a negative shift in the anodic peak potential with a slope of -0.0638 V. The slope obtained from Eq. (2) and its comparison with the theoretical value of -0.06 (m/n), where “m” is the number of protons and “n” is the number of electrons participating in the reaction, shows that the number of protons participating in the tyrosine oxidation reaction is equal to the number of electrons, in agreement with previous reports^37,38^.

2 $$ {\text{Epa}}\left( {\text{V}} \right) = - 0.0{638}\,{\text{pH}} + {1}.0{4}0{7}\quad \left( {{\text{R}}^{{2}} = 0.{9816}} \right) $$

**Figure 4:**
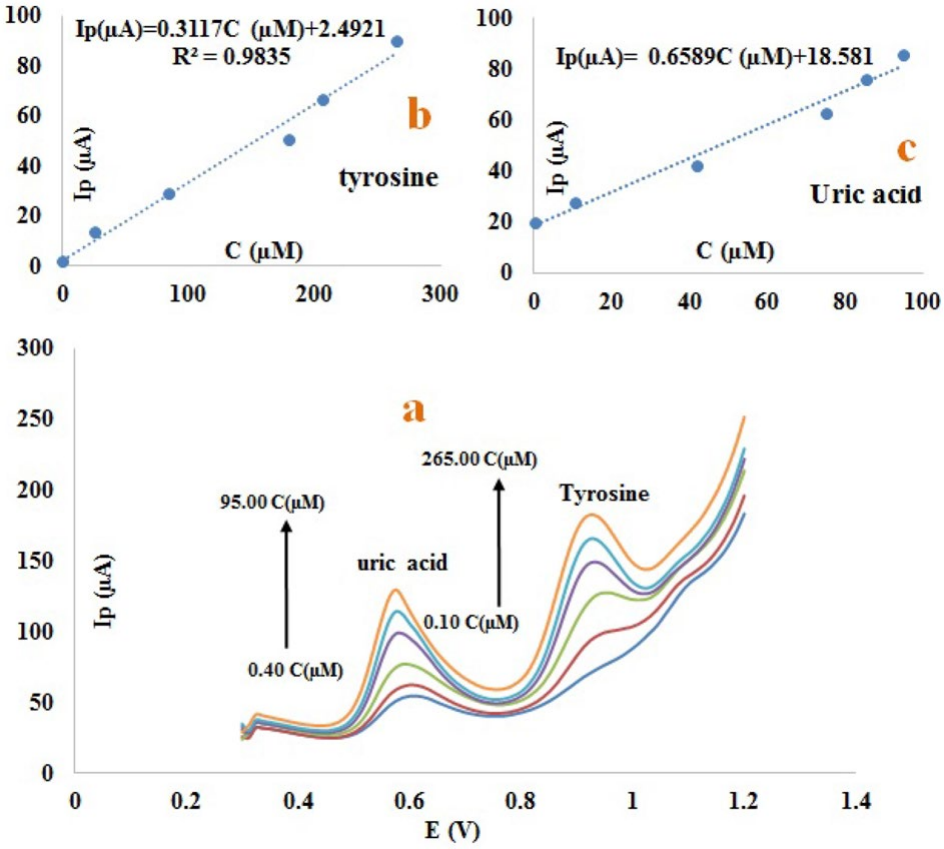
SWV of the human urine samples with different concentrations of uric acid(0.40, 10.49, 42.10, 75.40, 85.50, 95.00 µM) and tyrosine(0.10, 25.30, 83.70, 180.00, 206.00, 265.00 µM) at the surface of the modified electrode (**a**), the linear calibration curve of peak current versus different concentrations of tyrosine solution (**b**), the linear calibration curve of peak current versus different concentrations of uric solution Acid (c), in 0.2 mol L^−1^ phosphate buffer solution and 0.1 mol L^−1^ potassium chloride with pH 2.0. Electrochemical condition: pre-potential of 0.42 mv applied for 10 s, used electrodes (working electrode: modified carbon paste electrode, reference electrode: SCE, auxiliary electrode: platinum wire), temperature: 25 °C.

The oxidation peak current for tyrosine and uric acid is maximum at pH 2.0 and pH 3.0, respectively, and decreases as the pH of the current environment increases. These curves also show two peaks for tyrosine and uric acid, indicating two pka. For simultaneous determination of tyrosine and uric acid on the surface of the modified electrode, all voltammetric measurements were conducted in phosphate buffer solution with pH 2.0 as carrier electrolyte. On the surface of the modified electrode, the proposed mechanism for oxidizing tyrosine and uric acid can be seen in Fig. 4S-A,B.

### The effect of scan rate

As part of the investigation of the effect of potential sweep speed on uric acid and tyrosine electrochemical behavior on the surface of the modified electrode, the LSV was recorded for each of the solutions of uric acid and tyrosine in 0.1 mol L^−1^ phosphate buffer solution with pH 2.0, containing concentrations of 3.46 and 29.7 µmole L^−1^, respectively, with scanning rates ranging from 50 to 130 ms per second and 10–100 ms per second, respectively. The voltammograms (8-a) and (9-a) show that the anodic current increases with an increase in scanning rate, as can be seen in the voltammograms (Figs. 5S-a, 6S-a). Based on Figs. 5S-b and 6S-b as well as the results obtained from the analysis of the graph of changes in anodic peak current versus potential sweep speed, and the graph of logarithmic anodic peak current versus potential sweep rate in Figs. 5S-c and 6S-c, the mechanism of oxidation of uric acid and tyrosine is influenced by absorption, according to the results of the analysis.

### The effect of accumulation time

In each measurement, the modified electrode was immersed in a solution containing tyrosine and uric acid in order to study the effect of accumulation time on the anodic peak current. According to the results of this experiment, changing the accumulation time did not have any effect.

### The effect of pre-potential time

It also investigated whether the time at which the prepotential is applied affects the electrochemical response. An electrode with GO/AuNPs/MCPE modified was exposed to 0.42 mv of prepotential at different times in 0.1 mol L^−1^ phosphate buffer solution at pH 2.0 in the presence of 79.42 µmol L^−1^ uric acid. The square wave voltammetry technique showed an increase in the current of the electrode between 5 and 10 s, but no significant change was found beyond 10 s. It was therefore determined that 10 s would be the ideal time to apply the pre-potential (Fig. 7S).

### Electrochemical impedance spectroscopy (EIS) measurement

In order to investigate the surface resistance of modified electrode (GO/AuNPs/MCPE), the electrochemical impedance spectroscopy response in the presence of [Fe(CN)_6_]^−4/−3^ couple as a redox probe was evaluated using the unmodified electrode and modified electrode (Fig. 8S). All the optimized conditions are described in Table 1S.

## Measurement of carbon paste electrode surface

We measured electrode areas by LSV using [Fe(CN)6]^−4/−3^ as the probe in 0.2 mol L^−1^ phosphate buffer solution with pH 7.0 and different scanning rates. Randles–Sevcik equation is used for this reaction at T = 298 K.

3 $$ Ip = {2}.{69} \times {1}0^{{5}} {\text{n}}^{{{3}/{2}}} A\;{\text{D}}_{0}^{{{1}/{2}}} {\text{C}}_{0} *v^{{{1}/{2}}} $$

Equation (3) deals with the "anodic peak current," the electron transfer (n = 1), the electrode surface, the diffusion coefficient, the scanning velocity, and the concentration of Fe(CN)_6_^−4/−3^.

Using 0.07 µmol L^−1^ solution of [Fe(CN)_6_]^−4/−3^, the surface of the electrode in 0.2 mol L^−1^ phosphate buffer solution, T = 298 K, C_0_* = 7 × 10^−4^ M, D = 5.0810^−6^ cm^2^mol^−1^, n = 1, F = 96,485 Cmol^−1^, R = 8.314 jk^−1^ mol^−1^ was obtained. The surface area of the unmodified electrode and the modified electrode was 0.129 cm^2^ and 0.190 cm^2^, respectively, using Eq. (3).

### Repeatability

The square wave voltammograms of the modified electrode was performed using 4 consecutive tests using a concentration of 44.9 µmol L^−1^ uric acid and 45.3 µmol L^−1^ tyrosine solution in 0.2 mol L^−1^ phosphate buffer solution at pH 2.0 at a scanning rate of 100 mv/s for evaluating its repeatability behavior. Tyrosine had a relative standard deviation of 3.51% and uric acid had a relative standard deviation of 5.77%.

### Reproducibility

The same synthesis procedure was used to reproduce the oxidation current of three different modified electrodes (To prepare the electrode, gold nanoparticles-modified graphene oxide (8% w/w), graphite powder, and ecosan as an adhesive were mixed for 20 min at 40°C to obtain a homogenous mixture). Then, it was transferred to a Teflon tube with an inner diameter of 5 mm and an outer diameter of 2 mm, and its connection was established via a copper wire located in the middle of the Teflon tube) was calculated in uric acid and tyrosine solution with a concentration of 44.9 µmol L^−1^ and 45.3 µmol L^−1^ in 0.2 mol L^−1^ phosphate buffer solution, pH 2.0, and 0.1 mol L^−1^ potassium chloride at a scanning rate of 100 mv/s. Calculating the standard deviation, we found that tyrosine had a relative standard deviation of 5.13% and uric acid had a relative standard deviation of 8.42%, respectively.

### Calibration curve and Figures of merit

Following an evaluation of the parameters affecting the determination of tyrosine and uric acid, these two analytes were analyzed simultaneously, and a calibration curve was plotted. In order to accomplish this, uric acid and tyrosine solutions were prepared with different concentrations (0.06–141.00 µmol L^−1^ uric acid, 0.14–340.00 µmol L^−1^ tyrosine) in 0.2 mol L^−1^ phosphate buffer solution, pH 2.0, and 0.1 mol L^−1^ potassium chloride at a scanning rate of 100 mv/s. A SWV technique was then used to determine the optimal conditions for using the modified carbon paste electrode. Considering the high sensitivity of this method, the SWV method was used to determine the limit of detection and linear range for the simultaneous determination of both tyrosine and uric acid. Tyrosine and uric acid concentrations are clearly related to the anodic peak current and the peak current increases as tyrosine and uric acid concentrations increase. In Fig. 3, you can see the SWV for the simultaneous evaluation of tyrosine and uric acid. The concentration range of 0.14–340.00 µmol L^−1^ for tyrosine (Fig. 3b) and the concentration range of 0.06–141.00 µmol L^−1^ (Fig. 3c) for uric acid were obtained. The Method detection limit (MDL) of tyrosine and uric acid was obtained at 0.0060 µmol L^−1^ and 0.0037 µmol L^−1^, respectively, and the equation of the line for each of them is given as Eqs. (4) and (5) below.

4 $$ {\text{I}}\upmu {\text{A}} = 0.{39843}\;{\text{CM}} - {1}.{293}\quad {\text{R}}^{{2}} = 0.{9958} $$

5 $$ {\text{I}}\upmu {\text{A}} = {1}.{2813}\,{\text{CM}} - {3}.{4}0{23}\quad {\text{R}}^{{2}} = 0.{9844} $$

As a measure of the efficiency of the electrochemical method with the proposed electrode, the limit of detection and linear range obtained by this electrode were compared with some previously published literature (Table 1). In this comparison, it was found that the sensor prepared using the proposed method was highly efficient and cost-effective.

**Table 1:** Comparison of the proposed method with other methods.

Analyte	Analytical method	DLR (µM)	MLD (µM)	References
Tyrosine	DPV	30.0–150.0	0.01	^38^
	DPV	0.9–95.4	0.19	^39^
	DPV	0.1–400.0	0.046	^40^
	SPE	13.70–303.50	3.860	^41^
	Fluorescence	0.07–230.0	0.034	^42^
	Fluorescence	0.5–35.0	0.370	^43^
	SWV	0.14–340.0	0.006	This work
	DPV	0.01–100	0.032	^41^
Uric acid	SWV	0.60–100.0	0.13	^44^
	DPV	1.00–680	0.090	^45^
	UV–Visible	100.0–450.0	38.40	^46^
	HILIC	1.189–59.48	0.356	^47^
	SWV	0.06–141.0	0.0037	This work

### Application of the proposed sensor in real sample

Real samples were used to evaluate the efficiency and reliability of this MCPE. As part of this process, the urine sample was prepared in 0.2 mol L^−1^ phosphate buffer solution and 0.1 mol L^−1^ potassium chloride with pH 2.0, considering the DLR in simultaneous determination. Specific amounts of tyrosine and uric acid were added to the sample, and an SWV technique was utilized to record the voltammogram (Fig. 4a). The plot of concentration versus flow indicates that this method has a detection limit of 0.0067 µ moles L^−1^ and 0.001 µmole L^−1^, a correlation coefficient of 98.35% and 97.79%, and a sensitivity of 0.3117 and 0.6589 for tyrosine and uric acid, respectively, in real urine samples (Fig. 4b,c). Based on these results, the proposed method is applicable to the analysis of biological samples. Data for real samples are summarized in Table 2S.

### Investigating the possible interference effect of species

This method was tested in different matrixes in order to investigate its selectivity and efficiency. A quantitative study was conducted using solutions containing uric acid and tyrosine with concentrations of 0.014 mol L^−1^ and 0.07 mol L^−1^, respectively, as well as different concentrations of interfering species in pH 2.0 phosphate buffer solutions of 0.2 mol L^−1^ and 0.1 mol L^−1^ potassium chloride at Table 3S, the results confirm the feasibility of using the proposed method for both tyrosine and uric acid determinations.

## Conclusion

It is possible to use the modified GO/AuNPs/MCPE simultaneously to detect and determine tyrosine and uric acid. The potential difference between tyrosine and uric acid is 0.348 mv, allowing peak separation. This modified electrode shows high electrochemical activity for the oxidation of tyrosine and uric acid in the presence of other interfering compounds. Based on the electrochemical behavior of tyrosine and uric acid, the proposed modified sensor has a high sensitivity. As compared to previous studies, the proposed electrode exhibits a wide linear range in different concentrations and has a low limit of detection. The determination of tyrosine and uric acid in this research confirms the success of the suggested method. In the future, new materials can be used as electrochemical sensors to determine new compounds in complex matrices.

### Supplementary Information

Supplementary Information.

## Data Availability

The datasets generated and/or analyzed during this study are included in the article and supplementary material. More data are available from the corresponding author on reasonable request.
