# Supplementary material for: Decorated graphene oxide with gold nanoparticles as a sensitive modified carbon paste electrode for simultaneous determination of tyrosine and uric acid
Source: Sci Rep. 2023 Oct 15;13:17501. doi: 10.1038/s41598-023-44540-6 (PMC10577133; doi:10.1038/s41598-023-44540-6)
Supplement: Supplementary file 1 — Supplementary Information. [file 41598_2023_44540_MOESM1_ESM.docx]

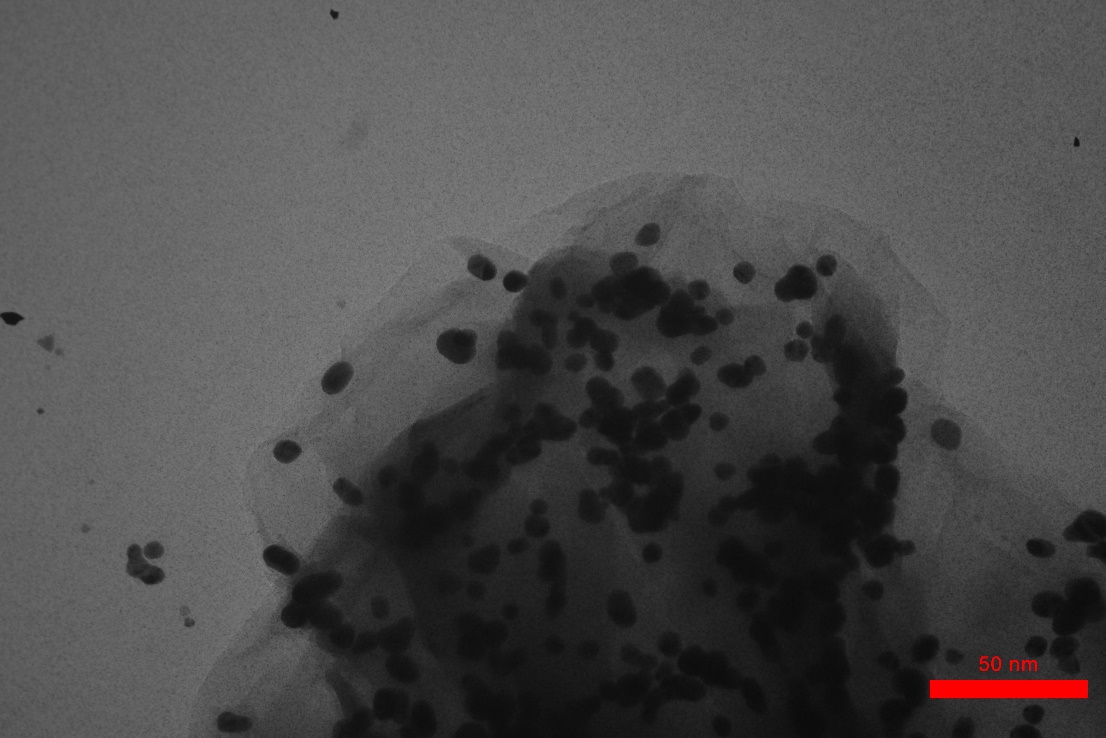


**Figure 1S.** TEM image of Graphene oxide-gold nanoparticles.


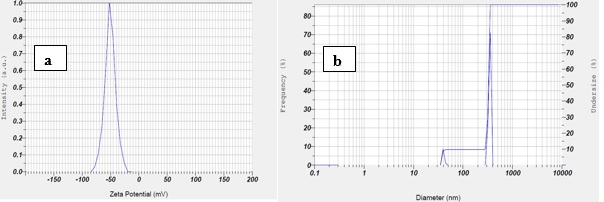


**Figure 2S.** (a) zeta potential and (b) Dynamic light scattering (DLS), zeta potential analysis of GO decorated with gold NPs hybrid nanofluid.


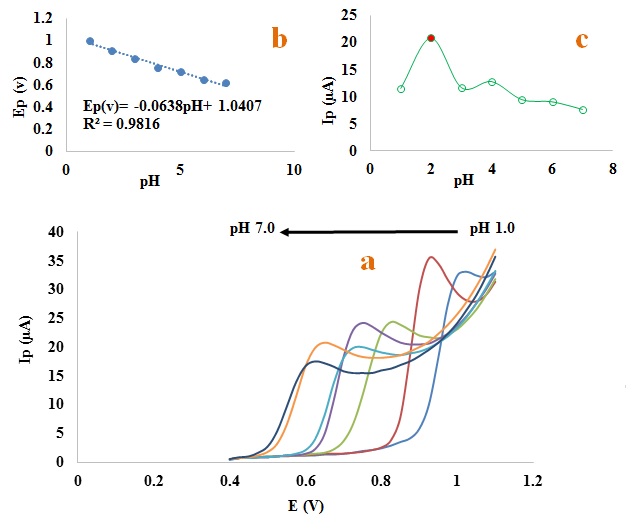


**Figure 3S.** LSV voltammogram of the oxidative behavior of 48.70 µmol L^-1^ tyrosine solution at different pHs (a), E_p_ vs. pH (b), Ip vs. pH (c) in 0.1 mol L^-1^ buffer phosphate solution with a scan rate of 100 mv/s, used electrodes (working electrode: modified carbon paste electrode, reference electrode: SCE, auxiliary electrode: platinum wire), temperature: 25 °C.

**Figure 4S.** The proposed oxidation mechanism of uric acid (A) and tyrosine (B) on the surface of the modified electrode.


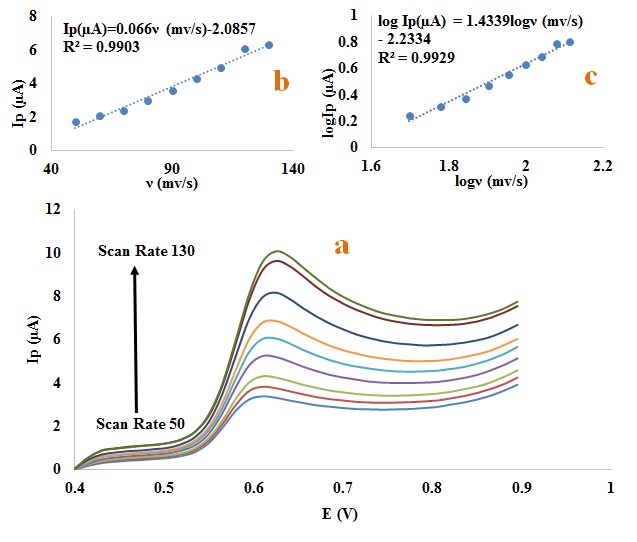


**Figure 5S.** LSV voltammogram of uric acid solution with a concentration of 3.46 µmol L^-1^ on the surface of the modified electrode (a), Ip vs. ν (b), log Ip vs. logν (c), in 0.1 mol L^-1^ phosphate buffer solution with a scan rate of 50-180 mv/s, used electrodes (working electrode: modified carbon paste electrode, reference electrode: SCE, auxiliary electrode: platinum wire), temperature: 25 °C.

**
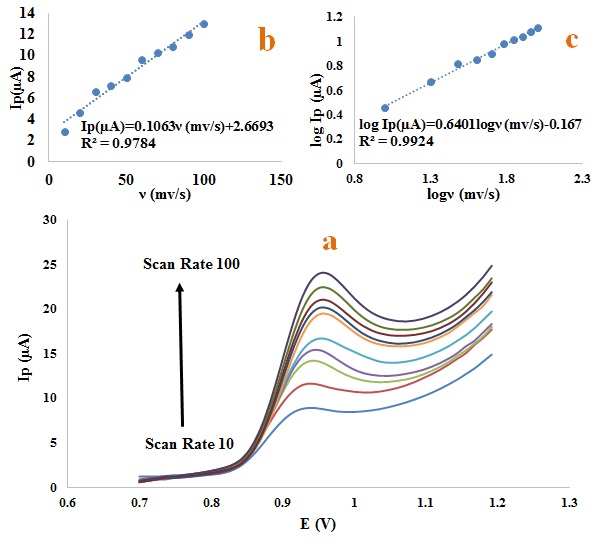
**

**Figure 6S.** LSV of tyrosine solution with a concentration of 29.7 µmol L-1 on the surface of the modified electrode (a), Ip vs. ν (b), logIp vs. logν (c), in 0.1 mol L-1 phosphate buffer solution with a scan rate of 10-100 mv/s, used electrodes (working electrode: modified carbon paste electrode, reference electrode: SCE, auxiliary electrode: platinum wire), temperature: 25 °C.


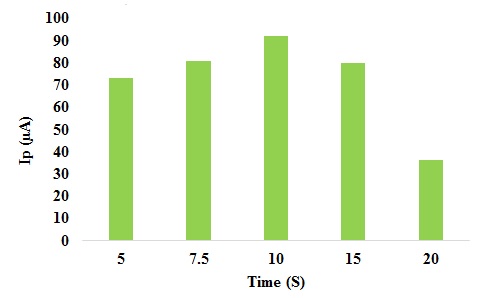


**Figure 7S.** The effect of pre-potential time on the response of the modified electrode in the presence of uric acid with a concentration of 79.42 µmol L^-1^ in 0.2 mol L^-1^ phosphate buffer solution with pH 2.0, using the SWV technique, used electrodes (working electrode: modified carbon paste electrode, reference electrode: SCE, auxiliary electrode: platinum wire), temperature: 25 °C.


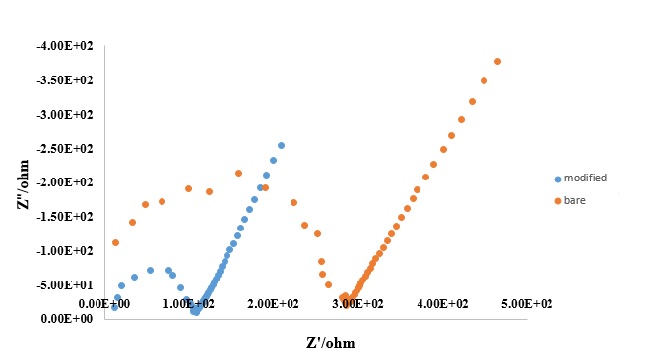


**Figure 8S.** Electrochemical impedance spectroscopy (EIS) of bare electrode (a), modified electrode (b) on surface CPE in 0.1 mol L^-1^ KCl containing 5.0 mmol L^-1^ [Fe(CN)_6_]^-4/-3^ frequency range: 0.1 Hz–100 kHz, amplitude: 10 mV.

| Petameter | pH | per potential value | per potential time | potassium chloride concentration |
| --- | --- | --- | --- | --- |
| optimum value | 0.2 phosphate buffer solution pH 2.0 | 0.42 V | 10 s | 0.1 M |

**Table 1S.** Optimized conditions of determination of tyrosine and uric acid via the electrochemical method

| Differences between actual value (µ) and mean value (M) | 1-sample t test  Pr = 0.05 | RR % | Found (Mean ± ts/√N)  N=3 and Pr ^c^ =0.05 | Added | molecule | Sample |
| --- | --- | --- | --- | --- | --- | --- |
| (µ = 25.00 and M = 25.01)  Not significant | P value = 0.955 | --- | ND ^b^ | NA ^a^ | Tyrosine | Urine sample |
|  |  | 100.04 | (25.01 ± 0.45) µM | 25.00 µM |  |  |
| (µ = 25.00 and M = 24.93)  Not significant | P value = 0.622 | --- | ND ^b^ | NA | Uric acid |  |
|  |  | 99.72 | (24.93 ± 0.35) µgM | 25.00 µM |  |  |

a: NA (Not Added)

b: ND (Not Detected)

c: Probability

**Table 2S:** The analysis of target molecules in real samples by the presented electrochemical sensor.

| **Foreign species** | **Interference level** | |
| --- | --- | --- |
|  | **Tyrosine** | **Uric acid** |
| Na^+^, K^+^, Cl^-^ | 60 | 60 |
| Ascorbic acid | 50 | 50 |
| L-glycine | 70 | 70 |
| L-methionine | 50 | 50 |
| L-cysteine | 30 | 30 |

**Table 3S.** Investigating the interference effect on the surface of the modified electrode in the presence of uric acid solution with the concentration of 0.014 µmol L^-1^ and tyrosine with the concentration of 0.07 µmol L^-1^ in 0.2 mol L^-1^ phosphate buffer solution and 0.1 mol L^-1^ potassium chloride, pH 2.0. Electrochemical condition: pre-potential of 0.42 mv applied for 10 seconds, used electrodes (working electrode: modified carbon paste electrode, reference electrode: SCE, auxiliary electrode: platinum wire), temperature: 25 °C.
